# Polycystic ovary syndrome and circulating inflammatory markers

**Published:** 2017-06

**Authors:** Farideh Zafari Zangeneh, Mohammad Mehdi Naghizadeh, Masoumeh Masoumi

**Affiliations:** 1 *Reproductive Health Research Center, Tehran University of Medical Sciences, Tehran, Iran*; 2 *Noncommunicable Diseases Research, Fasa University of Medical Sciences, Fasa, Iran.*

**Keywords:** Polycystic ovary syndrome, IL1α-1β, IL-17A, Sympathetic nervous system

## Abstract

**Background:**

Human and experimental studies suggest that the sympathetic regulatory drive in the ovary may be unbalanced (hyperactivity) in polycystic ovary syndrome (PCOS). Dysfunctional secretion of interleukin (IL) -1 (α & β) or related cytokines may thus be related to abnormal ovulation and luteinization.

**Objective:**

The aim of this study was the evaluation of cytokines’ pattern in PCOS women and discussion about the explanation of cross-talk between two super systems: sympathetic and immune systems and explanation sympatho-excitation and relationship with interleukins.

**Materials and Methods:**

In this study, 171 PCOS women aged between 20-40 years were studied the. Their body mass index was <28. The patients were divided into two groups: study group (n=85, PCOS women) and control group (n=86 normal women). The blood sample was obtained on the 3^rd^ day of menstruation cycle. IL-17, IL-1α, IL-1β, and TNF-α concentrations were determined in both groups.

**Results:**

The median serum level of IL-1α in the PCOS group was higher than the control group (293.3 and 8.0, respectively, p<0.001). Also, the median serum level of IL-1β was higher than the control group (5.9 and 3.1 respectively). But the median serum of level IL-17 in women with PCOS was significantly lower than the control group (p<0.001).

**Conclusion:**

Our results confirm that PCOS is a low-level chronic inflammation.

## Introduction

Polycystic ovary syndrome (PCOS) is the most common female endocrine disorder. Chronic anovulation, hyperandrogenism and polycystic in ovaries are the most common endocrine disorder in women between the ages of 18 and 44 (1). PCOS is a cause of female infertility and its prevalence is 15-20% among infertile women (2). Studies have been shown that PCOS is a chronic low-level inflammation and this chronic disease can be a potential cause of the long-term consequence of PCOS (3, 4). In vitro studies suggest that pro-inflammatory stimuli are capable of up-regulation of the steroidogenic enzymes for the production of androgens in theca cells of the ovary (hyperandrogenism) (5). ''This concept raises the possibility that inflammation may be capable of directly inducing hyperandrogenism in PCOS'' (6). 

52-64% of PCOS women have android obesity that is independently associated with the metabolic abnormalities like insulin resistant (IR) (7). Rahmouni and colleagues in 2004 reported that the intracerebroventricular injection of insulin increases sympathetic activation via the arcuate nucleus (8). The metabolic abnormalities like IR have a strong link with chronic inflammation (9). 

''Over the past decades, evidence has accumulated clearly demonstrating a pivotal role for the sympathetic nervous system and its neurotransmitters in regulating inflammation'' (10). In 1903 for the first time, the role of sympathetic nervous system in inflammation could be found in an article (11). Immunity barrier and immunoregulatory mechanisms are very important in the processes of follicle development, fertilization, and implantation of the fertilized egg in the uterus (12). The pro-inflammatory cytokines, particularly interleukin (IL)-1 activates the hypothalamous-pituotary-adrenal (HPA) axis (13). HPA axis controls adrenal steroidogenesis and metabolic factors including insulin and obesity-related signals (14). 

''IL-1 is a multifunctional cytokine and it has highly inflammatory features in reproductive biology and is believed to affect the processes of fertilization and implantation'' (15). Intra-cerebroventricular administration of IL-1 stimulates the production of Lutein hormone (LH) and follicle-stimulating hormone (1) from the gonadotropes (16). Interleukin-1 alpha and beta directly affect progesterone and oestradiol production in cultures of purified human granulosa cells (17). IL-1 alpha directly inhibites the production of oestradiol by human ovarian granulosa cells (18). IL-1β stimulates basal progesterone secretion in the human granulosa and theca cells and in small and large follicles in vitro (19). IL-1β in cumulus and granulosic cells of equine increases the level of PGF2-a and progesterone, which suggests that IL-1 can be involved in equine oocyte invitro maturation. (20). Gonadotropins can stimulate IL-1β and then it inhibits both LH and Human chorionic gonadotropin (LH/hCG) and FSH-stimulated progesterone and estradiol secretion by the follicular theca and granulosa cells, affecting cAMP production, that suggests a follicle-stage dependent regulatory role of IL-1 on ovarian follicles (21). Chen and coworkers demonstrated that the human granulosa-luteal cells express IL-1β transcript, and LH can stimulate this transcription in a dose-dependent manner. On the contrary, IL-1β significantly decreased LH-dependent estradiol production in these cells. ''These results suggest that LH may exert its action on the steroidogenesis of granulosa cell through, at least in part, the activation of the IL-1β gene'' (22). In the brain, interleukin-1β can reduce the release of monoamines: serotonin (5-HT), dopamine and noradrenaline. The role of IL-1β in the monoamine metabolism in the basal ganglia can help for plasticity of anxiety and depression in the brain (23).

17A is a prototypic member of the interleukin-17 (IL-17) cytokines. In this family, IL-17A, IL-17F, and IL-17E have known better. Two studies show that the role of these proteins in adaptive and innate immunity (24, 25). Experimental studies show that IL-17A has a novel potential role in the neuroanatomical plasticity of sympathetic autonome system SAS that can accompany inflammation (26, 27). 

The aim of this study was the investigation of the relationship between cytokines' (IL1α-1β, 17A, and tumor necrosis factor (TNF) pattern and SAS in women with PCOs. In the last few decades, PCOS is known as a chronic low-level inflammatory disease, so we need to know the pattern of these changes.

## Materials and methods

**Participants**

In this case-control study, 171 women were participated in two groups: 85 PCOS women (study group) and 86 healthy women (control group: male factor). is All women with PCOS visited at Vali-e-Asr infertility clinic affiliated to Tehran University of Medical Sciences from February 2012 to April 2013.. The European Society of Human Reproduction and Embryology and the American Society for Reproductive Medicine (ESHRE/ASRM) criteria were considered for diagnostic of women with PCOS (28). 

All of the participantsaged 20-40 yr without another chronic disease, for example,, immune system, cardiovascular, thyroid, diabetes and any medication. The body mass index (BMI) was under 28 and they have irregular menstrual cycle that is common in women with PCO. 5 ml venous blood sample was obtained from all participants on the third day of menstruation cycle. Serum samples for all patients were obtained by centrifugation at 3000 rpm for 10 min, then stored at -80^o^C until analysis. 

**Questionnaire**

A demographic questionnaire was filled for all participants. Clinical and anthropometric variables, including BMI, hirsutism and duration of infertility were recorded.

**Biochemical study**

The concentration of serum levels of IL-1α, IL-1β, IL-17 and tumor necrosis factor (TNFα) (ELISA kit, Monobind from Austria) were measured.

**Ethical consideration**

. Informed consent was obtained from all women. This study was approved in ethical committee of Tehran University of Medical Sciences as date and number: IR.TUMS.VCR.REC.1395.1329.

**Statistical analysis**

The mean and standard deviation was used for the quantitative variables and t test was applied for comparing between the study groups. The qualitative variables were presented with count and percentage and chi-square test was used for comparing between the study groups. According to departure from a normal distribution and for presenting inflammatory cytokines, the median was used. Mann-Whitney U test was used for variables between two study groups. The multinomial linear regression model was used to delet the confounding effect. The data were analyzed in IBM Statistical Package for the Social Sciences, version 19.0, SPSS Inc, Chicago, Illinois, USA (SPSS). P value less than 0.05 considered as the significant level. 

## Results

Weight and BMI were more in the PCOS group than the control group (p<0.001) Regarding the duration of marriage and infertility,parity and gravidity, there was no significant difference between two groups (Table I). But the occupation rate in the PCOS group was higher than the controls (p=0.002). The symptom of PCO was significantly higher in the PCO women. Serum level of IL-17α in the PCOS group was significantly lower than the control group (p<0.001). Both IL-1α (p<0.001) and IL-1β (p=0.017) were significantly higher in the PCOS than the control group (Table II). 

**Figure 1:**
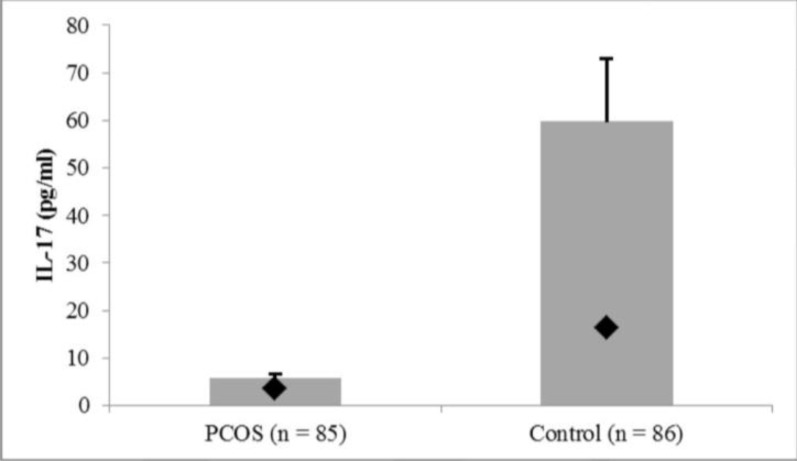
Comparison of mean and standard error (According to high departure from the normal distribution, SD makes the error bar very long, so SE has been used) of IL-17 between the control and PCOS groups. IL-17 in the PCO group was significantly lower than the controls. Point present median

A multinomial linear regression model was used to clarify the relationship between PCOS and IL17 (Table III). Age (β=-3.3, SEβ=1.4, p=0.020) and having PCOS (β=-58.8, SEβ=16.8, p=0.001) were significantly related with IL-17α (R2=0.139). In Figure 1, (Chart 1 was changed as figure 1), we showed that IL17α in the serum of women with PCO is lower than the control group.

**Table I:** Comparison of demographic data between the study and control groups

**Variables**		**Control group (n = 86)**	**PCOS group (n = 85)**	**p-value**
Age (2) †		29.5 ± 5.1	27.1 ± 4.4	0.001
Duration of marriage (2) †		6.88 ± 4.01	6.52 ± 3.42	0.537
Duration of infertility (2) †		4.33 ±3.96	4.84 ± 3.22	0.365
BMI (kg/m^2^) †		25.41 ± 3.76	27.39 ± 3.94	0.001
Gravidity (count) †		0.40 ± 0.69	0.41 ± 0.75	0.863
Years of education ‡				0.864
	Less than 12	27 (31.40%)	30 (35.29%)	
	12 (diploma)	46 (53.49%)	43 (50.59%)	
	More than 12	13 (15.12%)	12 (14.12%)	
Occupation ‡				0.002
	Housewife	14 (16.28%)	2 (2.35%)	
	Occupied	72 (83.72%)	83 (97.65%)	
menstrual state ‡				<0.001
	Regular	71 (82.56%)	27 (31.76%)	
	Irregular	15 (17.44%)	58 (68.24%)	

BMI: body mass index

† : Data are presented as mean ± standard deviation, using independent t- test

‡ : Data are presented as count (percentage), using chi-square test

**Table II:** Comparison of inflammatory cytokines between study and control groups

**Interleukin variation**	**PCOS group (n = 85)**		**Control group (n = 86)**		**P-value (Mann-Whitney)**
	**mean**	**SD**	**mean**	**SD**	
IL-1-α (pg/ml)	401.40	228.61	19.32	37.89	**<0.001**
IL1-β (pg/ml)	17.38	40.56	11.55	23.65	**0.017**
IL-17 (pg/ml)	5.80	6.90	59.92	121.11	**<0.001**
TNF-α (pg/ml)	10.91	26.42	45.60	172.48	0.119

SD: standard deviation.

IL-1-α;

IL1-β ;

IL-17 :

TNF-α:

**Table III:** Regression model of IL17 (Regression model R^2^ = 0.139

	**Unstandardized Coefficients**		**Standardized Coefficients**	**t**	**p-value**
	**B**	**Std. Error**			
Model constant	133.121	76.970		1.730	0.086
BMI (kg/m^2^)	1.316	1.782	0.060	0.738	0.462
Age (years)	-3.316	1.413	-0.190	-2.347	0.020
Occupation	-31.410	23.652	-0.106	-1.328	0.186
Irregular menstrual	-9.421	15.990	-0.054	-0.589	0.557
PCOS	-58.817	16.801	-0.341	-3.501	0.001

Age (P = 0.020) and PCO (P = 0.001) were significantly related with IL-17 .

BMI: body mass index

PCOS: Polycystic ovary syndrome

B: Regression coefficientsStd. Error: Standard Error

## Discussion

This study confirms that the PCOS is an inflammatory syndrome. The potential mutual interaction between two super systems (SAS and Immunity) can be the cause of increasing of serum levels of IL-1 in PCOS women. There are two issues for discussion of this study in central and peripheral areas: 1) Two axes, SAS and HPA axis in central as a neuroendocrine aspect that has a main pathologic background in PCOS, 2) the reflection effect of a neuro-immune-endocrine aspect of the ovary in PCOS.

Women with PCOS have significantly higher HPA axis and sympathetic nerve activity than their matched controls. The increased sympathetic outflow can be related to the hormonal and metabolic features that may be relevant to the pathophysiology of this syndrome (29). The pro-inflammatory cytokines, particularly interleukin-1 can activate HPA axis (30). Smagin and colleagues in 1996 showed that activation of the HPA axis is due to the noradrenergic terminals in the hypothalamus that play an important role in the IL-l induced (31). On the other hand, the noradrenergic innervation of hypothalamus can participate in the adrenocortical responses to the interleukin-1. Accordingly, of this response, noradrenaline (NA) increases the corticosterone-induced IL (32). ''IL-1 has been shown to up-regulate glucocorticoid receptor (GR mRNA expression in hypothalamic corticotrophin-releasing hormone (CRH) secreting cells'' (33). 

In women with PCO, cortisol level does not have significant changes (34). The up-regulation of GR can be the reason of normal rate of cortisol in women with PCO. These findings confirm Turnbull̕s report that the noradrenergic innervation of hypothalamus participates in responses of adrenocortical system to IL1. They suggest that ''this neuro-immune-endocrine action of cytokines is mediated primarily at the level of the central nervous system (CNS)'' (13). 

In CNS, some nuclei of the supraspinal brain, such as collection of nuclei in the hypothalamus, locus coeruleus (LC) and amygdala which are Trans neuronal connected with the ovary. All these nuclei are involved in the physiologic function of the ovaries. LC is the major noradrenaline NA nucleus in the brain. LC located adjacent to the IV ventricle in the pontine brainstem. Anselmo-Franci and coworkers, in 1997 showed that the electrolytic lesions of the LC can block the pre-ovulatory surge of LH in the ovary of the rat. The reduction of NA in the medial basal hypothalamus (3) is the main factor for blockade of LH surge (35). Because the positive feedback action of estradiol (E_2_) on LH secretion is the most important role of LC in central function of the reproductive system (36). Our previous study showed that LC lesion could augment the estradiol concentration in PCO rats. This finding suggests that blocking the release of NA by LC lesion in rats with PCO can increase the serum concentrations of estradiol (37). 

Raphael *et al* also in 2013, reported that the stimulatory effect of NA on the release of gonadotropin-releasing hormone occurs during the positive feedback of ovarian steroids (38). Estrogen up-regulates NA release in the Medio basal hypothalamus. Increased levels of estrogen from the ovaries can be the primary cause of the Gonadotropin releasing hormone surge for triggering of Luteinizing hormone surge (39). 

This positive correlation between serum estradiol levels and NA, in patients with PCOS, can be the main executive factor at the brain-ovary axis. All of these findings confirm the main role of SAS (NA) on ovary function and dysregulation of it on the brain-ovary axis in PCOS.

Inflammation is not only an acute response to trauma or infection, but it is also a response to the ongoing processes of cell turnover associated with aging. In this regard, the inflammatory response regulates fundamental processes intrinsic to cellular homeostasis, including proliferation, necrosis, and apoptosis (40). ''The progression of apoptosis in follicular cells is dependent on the cooperative regulation of different paracrine and autocrine factors; it is likely that none of these factors are specifically required in the control of follicle growth or death'' (41). One of the extrinsic factors as initiating mechanisms of apoptosis in follicle atresia process is cytokines (42). The present study demonstrates that the crosstalk between these two super systems, SAS and immune system in women with PCOS. The inflammation amplifier is activated by NA and the stimulation of cytokines, such as TNF-α, IL-17 in the subsequent expression of various target genes for chemokines (43). 

Petit and colleagues reported that ''the enhanced whole body glucose metabolism seen after central administration of IL-1α is mediated by increased sympathoadrenal activity. They suggested that the IL-1α induced increase in pancreatic insulin and glucagon secretion, as well as part of the peripheral catecholamine release,, is mediated by central adrenoreceptors'' (44). In the other hand, the administration of systemic and intracerebral IL1 can increase the turnover of NA in hippocampus and hypothalamus in the brain (45). McNamee and coworkers, in 2010 suggested that NA has neuroprotective properties because negatively regulates the IL-1 system. This neuro-inflammation is characterized by β-adrenoceptor (46). These findings confirm the important role of SAS in the neuro-immune aspect of PCOS. So, the over activity of SAS can never play the normal negative regulatory role for reduction of IL1 in women with PCOS. PCOS is a chronic condition and recent studies show that PCOS as chronic low-grade subclinical inflammation which has been increasingly recognized as an interposer in the endocrine, metabolic and reproductive disturbances. 

In this study, the inflammatory nature of PCOS (low-grade chronic) was confirmed as similar reports over the past decade (47). ''Sympatho-adrenergic pathways in the innate immune system may represent novel anti-inflammatory and immune-modulating targets with significant therapeutic potential'' (48). The crosstalk between two super systems is clear but in PCOS women the role of NA in mediating inflammatory responses is less clear, with evidence of both pro- and anti-inflammatory effects. 

In this study, we suggest that in women with PCOS: 1) Increased IL-1α can impair the feedback system of NA (neuro-inflammation process) or vice versa. these data confirm Luotola̕s study in 2016 that explained an increased IL-1Ra level in women with PCOS (49). 2) Increased IL-1β could be due to the lack of ovulation (anovulation) in women with PCOS (50). 3) Regulation of IL-17A by adrenal hormones caused a significant reduction of this cytokine in the serum of PCOS women that confirms the result of experimental study of Bosmann and coworkers, in 2013 (51). ''IL-17 can act on sympathetic somata and distal neurites to enhance neurite outgrowth and identify a novel potential role for IL-17 in the neuroanatomical plasticity that accompanies inflammation'' (52). 4) This impaired cytokine pattern possibly can play a major role in the immunopathogenesis of PCOS. 5) The investigation of the neuro-immune axis can be an effective study in identifying the etiology of PCOS.

## Conclusion

This study confirms that the PCOS is an inflammation disease with an abnormal lymphocyte subset hat possibly associated with a dysfunction in the neuro-immune axis. 
